# Navigating Agricultural Expansion in Harsh Conditions in Russia: Balancing Development with Insect Protection in the Era of Pesticides

**DOI:** 10.3390/insects14060557

**Published:** 2023-06-15

**Authors:** Dmitry Zharkov, Timur Nizamutdinov, Dmitry Dubovikoff, Evgeny Abakumov, Alena Pospelova

**Affiliations:** 1 Department of Applied Ecology, Faculty of Biology, Saint Petersburg State University, Saint Petersburg 199034, Russia; t.nizamutdinov@spbu.ru (T.N.); d.dubovikoff@spbu.ru (D.D.); e.abakumov@spbu.ru (E.A.); 2 Department of Invertebrate Zoology, Faculty of Biology, Perm State National Research University, Perm 614068, Russia; alena.pospelova18@mail.ru

**Keywords:** agroecosystems, pesticides, biodiversity, insect populations declining, plant protection, sustainable agriculture

## Abstract

**Simple Summary:**

Insects play a crucial role in the functioning of terrestrial ecosystems. Chemical pest control methods continue to dominate in addressing food security issues in Russia. However, competent research on the impact of pesticides on non-target insect species is critically lacking both globally and in Russia. The article highlights the challenges of studying the effects of pesticides on insects and the vulnerability of insects to pesticides in regions with harsh conditions. It calls for the development of new sustainable agricultural practices to ensure coexistence between pest control and sustainable development. Furthermore, the article emphasises the importance of a legal framework regulating pesticide use and discusses new successful methods of sustainable farming. These findings contribute to the progress of the research discipline by promoting sustainable agricultural practices and striking a balance between agricultural expansion and insect protection. The focus on the significance of sustainable agriculture in harsh conditions also has implications for other regions facing similar challenges.

**Abstract:**

As the world’s population continues to increase, ensuring food security becomes a major problem. This often leads to the expansion of agricultural production, even in harsh conditions and becomes a key problem for many countries, including Russia. However, such expansion may entail certain costs, including the potential loss of insect populations, which are vital for ecological balance and agricultural productivity. The development of fallow lands in these regions is necessary to increase food production and increase food security; it is important to balance this with protection from harmful insects and sustainable farming methods. Research into the effects of insecticides on insects is an ongoing challenge, and new, sustainable farming methods are needed to ensure that protection from harmful insects and sustainable development can coexist. This article discusses the use of pesticides to protect the well-being of mankind, the problems of studying the effects of pesticides on insects and the vulnerability of insects to pesticides in regions with harsh conditions. It also discusses successful methods of sustainable agriculture and the importance of the legal framework governing the use of pesticides. The article emphasises the importance of balanced development with insect protection to ensure the sustainability of agricultural expansion in harsh conditions.

## 1. Introduction

Insects play one of the most important roles in the functioning of the biosphere [1]. In terms of species diversity, they outnumber all groups of living creatures. Scientists have now described more than one million insect species, more than all other groups combined [2]. Currently, thousands of new species are described each year [3]. Insects are integral to both the energy and biogeochemical cycles of chemical elements, and have extensive ecological relationships with other organisms and the environment. As a result, they play a crucial role in establishing regulatory and stabilising mechanisms within ecosystems [4]. They are also the main pollinators of most wild and cultivated flowering plant species [5]. Currently, numerous authors have observed a reduction in both the diversity and population of insects, with agricultural expansion and intensification being widely regarded as the primary contributing factor in most studies [6,7,8,9,10,11]. Various estimates of reductions in biodiversity and insect abundance in different groups can be as high as 90 percent [12,13,14,15,16].

According to the most conservative estimates, only one fifth of the insect diversity on the planet has been described to date [17]. Consequently, the importance of insects cannot be objectively or fully evaluated, let alone quantified. It is challenging for these enigmatic “assets” to capture the serious attention of the public and policymakers, given the apparent paucity of information regarding them. It is an established fact that human activity can cause substantial damage to natural systems, and it is also acknowledged that this harm can have adverse effects on human welfare. This problem becomes more evident when using the concept of ecosystem services. Ecological services refer to the benefits and/or profits derived from the use or non-use of ecosystems that directly or positively contribute to human welfare [18,19]. These services fall into four major groups: regulating, supporting, providing, and cultural. The most important ecosystem services provided by insects are pollination (regulating), pest control (supporting), and participation in the nutrient cycle (providing) [20,21,22]. Insects and their products also provide resources for organisms at a higher trophic level, including humans [20,23]. They also form a major part of the food sources for birds, reptiles, fish, and many other vertebrates [24]. Meanwhile, the ecosystem services associated with insects are grossly underestimated. An example is a number of cultural services (including recreational components). A number of insects, primarily butterflies, large beetles, and a hymenopterans (such as ants and bumblebees, for example) have important aesthetic significance and attract the attention of people when relaxing in nature. A number of such species are quite common even now in the North. A few of them are included in the Red Book of the Russian Federation and regional lists as having aesthetic value, especially attractive great colonies of red wood ants (*Formica* s. str.) with big and numerous mounds.

Nonetheless, there are certain insect species that can have a detrimental impact on human health and well-being by carrying harmful pathogens or causing extensive damage to crops [22]. Such services are often referred to as anti-services or disservices [25]. In natural ecosystems that are not influenced by human activities, the abundance of carrier and harmful insect species is regulated by a multitude of interactions within food chains and the conditions of their habitats. However, in agroecosystems, disturbances to the delicate balance of these interactions, such as deforestation, habitat fragmentation, and intensive agricultural practices, can modify them and result in outbreaks of these harmful species [26,27]. An agroecosystem is developed and overseen by humans, utilising the intricate natural ecosystems, with the objective of achieving the highest possible crop yield. Adverse phytosanitary conditions of agrobiocenoses can lead to significant losses, and with the combined negative impact of pests, pathogens, and weeds, up to 50% of the crop yield can be lost [28]. Various chemicals (pesticides and fertilisers) are used in agroecosystems to increase productivity and to suppress the activity of pests, which have an impact on the environment. Substances used to suppress harmful insects (insecticides) can have a negative impact on other arthropods, including “beneficial” ones such as pollinators, pest-feeding predators, and soil biota. Excessive use of such insecticides can lead to a reduction in the abundance and biomass of non-target insects both locally and globally, and in combination with other factors such as massive monoculture, habitat alteration, and climate change, it will have far-reaching consequences at the community level. The loss of insect biomass, abundance, and diversity will disrupt trophic cascades, leading to the decline of flowering plants and alteration of terrestrial food webs [29].

In the near future, the population of Russia and Eurasia as a whole may face the need to expand the agricultural lands to more northern regions, which is due to both climate effects and the global restructuring of agrotechnical practices in the Northern Hemisphere [30]. The northern regions can be considered “harsh conditions” for farming for several reasons: firstly, polar regions are known for their extremely cold temperatures, especially during winter. The Arctic Circle, for example, experiences sub-zero temperatures for a significant portion of the year. Such extreme cold can pose challenges for both plants and animals, as they need to adapt to survive in these freezing conditions. Secondly, during winter in polar regions, there are long periods of darkness where the sun does not rise above the horizon. This lack of sunlight limits the availability of solar energy for photosynthesis, which is essential for plant growth. Thirdly, polar regions have a short growing season due to the prolonged winter and limited period of favourable conditions for plant growth. This limited time frame restricts the types of crops that can be grown and requires careful planning to maximise productivity within a short period. Fourthly, permafrost is a layer of permanently frozen ground found in polar regions. It poses challenges for agriculture because it restricts the depth to which roots can penetrate and limits water drainage. Melting permafrost can also lead to uneven ground and soil instability. Finally, polar regions are typically remote and have limited access to transportation and infrastructure. The lack of roads, paths, and other facilities can hinder the transportation of equipment, supplies, and harvested produce. This logistical challenge adds to the complexity of farming operations in these regions.

To expand agriculture in these harsh conditions, it is also necessary to take into account the vulnerability and resilience of these ecosystems, since due to low biological diversity, they are often less stable and more susceptible to degradation under anthropogenic pressure, including agricultural practices [31,32]. The use of pesticides can further exacerbate this instability, leading to a decline in populations of beneficial insects and other non-target species. As a result, the delicate balance of these ecosystems will be disrupted, and there will be a negative impact on the ecosystem services they provide, which could be reduced to zero or even transformed into disservices (the opposite of ecosystem services). This is likely to lead to a decrease in crop yields and a reduction in the overall health and productivity of the agricultural system. Thus, the productivity of the new acreage will not reach the level in areas more suitable for agriculture. To ensure the sustainability of agricultural production in harsh conditions, it is important to balance the need to protect agricultural crops with the need to protect the ecosystem and its biodiversity. This requires the development and implementation of comprehensive strategies for pest management, prioritising the use of non-chemical methods and minimising the use of pesticides. By taking a holistic approach to agriculture in harsh conditions, it is possible to promote both agriculture and insect control while preserving the health and integrity of the ecosystem.

The aim of this article is to explore the challenges of balancing agricultural expansion with the protection of insect populations in harsh conditions, particularly in the context of pesticide use. To achieve this aim, the following objectives were set:

(1) To discuss the use of pesticides as a means of protecting the well-being of mankind;

(2) To explore the challenges associated with studying the effects of pesticides on insects and the importance of continued research in this area;

(3) To discuss the vulnerability of non-target insects to pesticides in the context of expanding agricultural production in harsh conditions;

(4) To discuss new, successful methods of sustainable agriculture;

(5) To emphasise the importance of legal regulations and frameworks governing the use of pesticides in ensuring sustainable and responsible agricultural expansion.

## 2. Are Pesticides Always the Best Way to Guard the Well-Being of Mankind?

In 2022, the world population exceeded eight billion people and is projected to reach almost 10 billion by 2050 [33]. In order to feed the growing population, agriculture is becoming increasingly intensive: mass monoculture farming, excessive use of fertiliser pesticides (particularly herbicides, insecticides, and fungicides), and the destruction of natural insect habitats on farmland and surrounding areas, are all aimed at increasing productivity [34]. However, this has serious negative global side effects, such as the loss of natural habitats for animals and especially rapid declines in insect biodiversity [33,34]. Therefore, the development of a scientifically-based sustainable approach to agricultural intensification should take precedence.

The global use of pesticides has increased over the last 20 years to 3.5 billion kg per year, representing a global market worth $45 billion [35]. According to BusinessStat [36] estimates, the production of plant protection chemicals in Russia increased by 90.1% from 45.3 thousand tons to 86.1 thousand tons from 2014 to 2018, and from 2017 to 2021, pesticide production in Russia increased by 1.8 times from 86.8 thousand tons to 155.6 thousand tons. The amount of insecticides in the period from 2014 to 2018 ranged from 9.7% in 2017 to 12.7% in 2016 (12.1% in 2018). Undoubtedly, the chemical method of plant protection continues to dominate in addressing food security issues in Russia [28].

One of the positive aspects of using insecticides is the fight against transmissible diseases, where the most effective means of control is often the destruction of the carriers. Insecticides are often the one of the most practical ways to combat insects that spread deadly diseases such as malaria, which kills around 5000 people worldwide every day [37]. Insecticides also protect buildings and other wooden structures from damage by termites and carpenter millers.

Ideally, an insecticide should be lethal to target pests but not to non-target species, including humans. Unfortunately, this is not always the case, as the effect of translocation occurs when these substances enter the human body through food, raising questions about the rational use and overuse of insecticides. Species that are highly dependent on specific environmental conditions suffer, while species with high ecological plasticity become pests, resulting in a significant decrease in insect biomass. Although rare species may have relatively small functional importance (in terms of contributions to ecosystem services such as pollination, pest control, etc.) compared to common species, they typically have high conservation value [38,39]. In addition, the widespread and irrational use of insecticides leads to the emergence of pests that are resistant to these substances. For example, *Leptinotarsa decemlineata* (Say, 1824) has developed resistance to almost all insecticides used against it [40]. To control this, new substances need to be developed. This not only entails huge additional financial costs, but also additional negative impacts on the environment. In fact, we are in an “arms race” with nature.

## 3. Problems in Research on the Effects of Insecticides on Insects

Over the past 30 years, the impact of insecticides on non-target arthropod species has been the subject of numerous studies worldwide, and potential consequences have been considered repeatedly [e.g., [41,42,43,44]; In Russia: [45,46,47,48,49,50,51]]. Existing studies on the impact of insecticides on insects have often included changes in physiological and biochemical processes, both in pollinator models and in natural enemy models in laboratory conditions [e.g., [52,53]].

A major issue may be that laboratory conditions cannot reflect real processes in nature, as they assume strict selection based only on one factor (the impact of insecticides) while weakening it for all other factors (predators, parasites, diseases, adverse weather conditions, etc.) and without taking into account the synergistic and antagonistic effects of these factors. More in-depth studies have only been conducted on honey bees, as they have economic value, while others have been ignored, such as over 20,000 species of bees. For this reason, several countries have special service governmental decrees for the protection of honey bees (e.g., as a result, in several countries, special government regulations are in place to protect honeybees. For example, in Germany, the National Bee Monitoring Programme: The Federal Agency for Nature Conservation (BfN) has been established to collect data on bee populations and their health. Additionally special governmental decrees on bee conservation have been issued (Denmark, Austria, Italy, France, Germany etc.). Further, natural enemies of pests receive significant attention due to their value in integrated pest management [e.g., [54,55,56,57,58]].

There is not enough research (including in Russia) on the impact of insecticides on non-target insect species in field conditions, and they show conflicting results from study to study. However, these studies may encounter several problems when interpreting data. For example, studying insect biomass per unit area by season may not reflect the real state of the ecosystem for several reasons. Several potential factors may lead to temporal autocorrelation in detection probability, suggesting that the population size changes over time as individuals become more or less detectable [14,59]. This “detection effect” can be triggered if the ability to detect or count individuals varies depending on external factors (environmental conditions, anthropogenic impacts), regardless of their actual population size. In addition, the most obvious reason could be that the level of insect activity (and therefore the probability of detection) depends on certain weather conditions in the environment, which also vary over time.

Another factor could be the potential sublethal effects, which refer to the physiological or behavioural impact on individuals exposed to pesticide (where the dose/concentration may not be lethal but sublethal). Sublethal consequences can manifest in physiological and behavioural changes. These effects can also introduce biases in data regarding actual population size due to alterations in detection or counting frequency. Detection can also vary with the increase or decrease of human influence on other aspects of the environment over specific time intervals, such as artificial nighttime lighting [60].

Some insect species may develop resistance to insecticides, and there may be a lack of long-term data on the impact of insecticides, which can hinder the determination of the true effects of these chemical substances on insect populations.

It should also be mentioned that many studies on insect biomass are based on the assumption that insect dispersal rate is independent of their density. Most insect monitoring methods rely on counting the number of moving insects in traps. Logically, this implies that changes in sample size are a good indicator of population size changes only if activity rates are not dependent on density. The problem is that at high population densities, one would expect an increase in dispersal rate (e.g., due to resource limitation), while at lower densities, a decrease in dispersal rate could be expected, and this relationship may not be linear [61,62].

Movement-based monitoring methods can thus overestimate insect abundance at the peak of actual population size and underestimate population size during periods of decline, potentially leading to overestimation of population decline rates as populations decrease (and vice versa as populations increase). Furthermore, these changes can vary among different insect groups.

Lastly, local consequences of population decline may not reflect broader regional changes in population size, as the initial response of insects to environmental conditions is often local migration. For this reason, distortions may occur due to low recruitment rates at specific sample points [60].

The interconnections within natural systems are so complex that it is impossible to predict all the consequences of changes in these systems without specialised studies that consider the aforementioned factors. The primary focus should be on preventing adverse impacts on the environment and employing methods that are both environmentally and economically sustainable.

## 4. Vulnerability of Non-Target Insects to Pesticides in the Context of the Development of Regions with Harsh Conditions as a Model of Agricultural Expansion

The issue of food security in the world is becoming increasingly acute each year [63]. The most fertile lands are already being intensively utilised, while many countries are faced with the challenge of significantly increasing food production to meet the needs of their rapidly growing populations [33]. The need to increase food production and the limited availability of fertile land have led to the expansion of agriculture in harsh conditions, including regions in Russia.

Polar agriculture (as a scientific approach to the cultivation of food crops under extreme conditions) began to develop in Russia in 1917. The intensification of agricultural development of the northern territories during the Soviet period was primarily related to the supply of the constantly growing population of remote areas with food, since the transportation of products to far away areas was difficult, and some of them fell into disrepair during long-term transportation [64]. Up to that point, only 1.5% of the country’s cropped area was above the latitude of St. Petersburg (59°57′) [65]. During the 1920s and 1930s, intensive research of ways to develop the northern territories was started, which was based on the scientific approach [65]. Work was initiated to organize a system of experimental agricultural facilities in the north, where scientists and agronomists were engaged in the development of new varieties of crops. The network of agricultural stations was quite extensive; we give the locations of some of them: Khibinskaya agricultural station, (Murmansk region, Russia, 67.54 N, 33.37 E), Naryan-Marskaya experimental agricultural station (Nenets Autonomous Okrug, Russia, 67.63 N, 52.98 E), Vorkutinskaya station (Komi Republic, Russia, 67.50 N, 64.07 E), Yamalskaya zonal experimental station (Yamalo-Nenets Autonomous Okrug, Russia, 66.52 N, 66.59 E), Verkhoyanskiy base station (Republic of Sakha (Yakutia), Russia, 67.54 N, 133.40 E) and many others [66]. In 1937, the Research Institute of Polar Agriculture and Animal Husbandry (Norilsk, Krasnoyarsk Krai, Russia) was organized (69.3420, 88.2025). Thus, the network of scientific institutions covered geographically almost the entire Russian Far North. Vavilov and Eichfeld proposed a zonal concept for the development of polar agriculture. In the first zone (geographically the Arctic tundra zone) it was proposed to develop greenhouse farming; in the second zone (forest tundra zone and the southern border of tundras), the open-field cultivation of greens and root crops (radishes, turnips, potatoes, etc.), as well as the development of greenhouse vegetable farming. In the third zone (northern taiga, southern border of the forest tundra), open field cultivation of oats, barley, rye, flax, cabbage, and potatoes was planned [67,68,69,70]. The main factors limiting the development of agriculture in the far north were: low temperatures, short duration of the period available for crop development (from 40–45 to 100–105 days), extremely low fertility of zonal soils, and lack of moisture (drought) in some areas [71].

**Figure 1:**
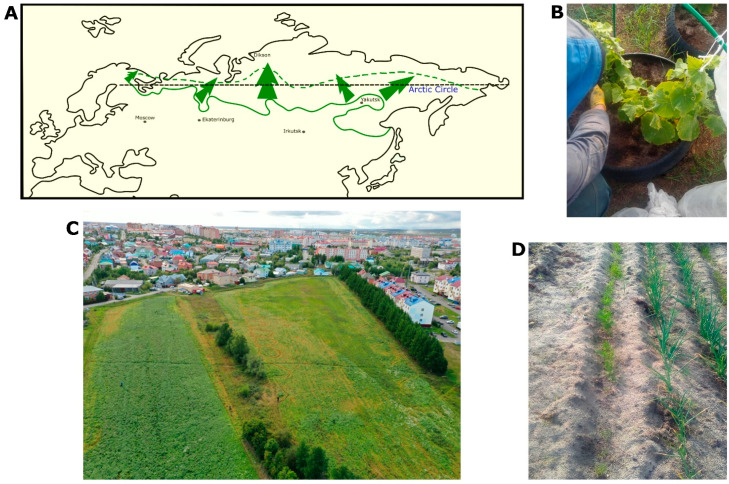
(**A**)—Schematic movement of the agricultural border to the north (redrawn from Mikhailov, 1947 [65]); (**B**)—Cucumbers in the vicinity of Nadym city; (**C**)—Yamal Experimental Station Field located on polar circle, Salekhard; (**D**)—Carrot and onion in the vicinity of Nadym city (65.32 N, 72.31 E).

After the collapse of the USSR, a significant portion of agricultural land was designated as fallow or unused. The 2016 Agricultural Census, conducted in accordance with Resolution No. 316 of the Government of the Russian Federation of 10 April 2013 “On the organisation of the 2016 All-Russian Agricultural Census”, showed that the total area of unused agricultural land in Russia was 97.2 million ha –44% of all agricultural land in the country. In 2021, the Ministry of Agriculture of the Russian Federation adopted a state program called “Efficient Utilisation of Agricultural Land and Development of Land Reclamation Complex in Russia” (commonly referred to as the “Second virgin land”) [72]. The development of these lands should be carried out strictly on a scientific basis, taking into account the specific conditions of the regions (Figure 1). It is important to regulate the use of pesticides and ensure that they are used only when necessary and responsibly, taking into account their potential impact on the environment and non-target organisms such as bees and other pollinators. Additionally, the protection and preservation of biodiversity, including insects, should be a priority in the development of agriculture in harsh conditions.

**Figure 2:**
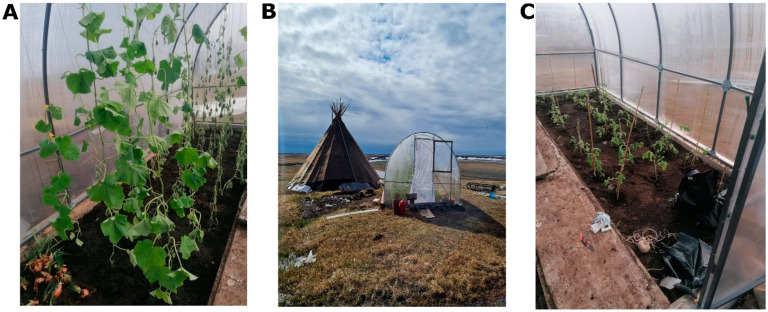
Greenhouse cultivation of cucumbers and tomatoes in the far north, Seyakha settlement (degree north latitude). (**A**)—cucumbers; (**B**)—the chum; (**C**)—tomatoes. F.

Similarly, the current climate change, which affects the Arctic region to a greater extent, holds a special significance as these changes are felt there much more intensely than on average across the Earth [73,74,75]. Over the past decades, the Arctic coastline of Eurasia has experienced a temperature increase of 2–3 °C. The degradation of the permafrost soil complex within the permafrost zone potentially opens up opportunities for expanding land use and pushing it further northward. Currently, the development of the Arctic is progressing at an accelerating pace, as it represents one of the strategic priorities of the Russian Federation. The use of specialized farming practices made it possible to achieve very high yields under harsh conditions. First of all, it is necessary to increase the fertility of zonal soils; in mineral soils, fertility regulation is achieved by applying large doses of organomineral and organic fertilizers (doses up to 150 t/ha) [71,76,77,78]. There is a “construction” of new soils—anthrosols, which have the required fertility parameters. As a result of introducing huge quantities of organic matter, the thickness of organomineral horizons can rise from 3–5 cm to 20–30 or even 40 cm [66]. Liming of soils to reduce their acidity and drainage during agricultural development of swampy areas is also necessary. Such activities in the zonal soil cover also lead to a decrease of the permafrost depth, so in the mature tundra the depth of permafrost is 20–30 cm, in newly developed areas it is reduced to 120–150 cm, and in the old tillage areas to 200–250 cm [69,79]. An important role in the organization of polar open-field farming is played by the organization of windproof forest (or shrub) stands around the fields, and the way organization of snow accumulation works (to prevent deep soil freezing during the winter period), which is also very important, ensuring rapid snowmelt (usually, snow-melt is enhanced in the fields by spraying coal dust) [76]. There are also special practices of greenhouse farming common to local residents and agricultural enterprises (Figure 2). To regulate the temperature regime and reduce the impact of low temperatures on the crop, greenhouses are placed above the soil cover (at a height of 15–30 cm) or the bottom of greenhouses is insulated with special materials with low thermal conductivity. For example, in Tovopogol village, (66.5208, 68.0098) locals, applying such farming practices and using special fish composts, grow vegetables (cucumbers and tomatoes) and even berries (strawberries) in a rather short vegetation period. The use of special composting technologies also makes it possible to achieve impressive soil fertility parameters in open ground and in greenhouses [77].

As a result, the anthropogenic impact on the unique and vulnerable ecosystems of the permafrost zone is intensifying. The region is highly sensitive to climate fluctuations and anthropogenic pressures due to its low biodiversity, which requires heightened environmental security measures [80,81]. Climate change is not only a scientific or ecological problem but also, to a much greater extent, an economic one [82]. The resolution of these issues directly impacts the socio-economic and political interests of Arctic countries. Undoubtedly, the Russian Federation plays a leading role in this regard, as it possesses a significant portion of the region, and climate change creates a situation that necessitates an urgent, comprehensive, and balanced national approach to climate and related issues. This approach should be based on thorough scientific analysis of environmental, economic, and social factors. Research in high-latitude regions of Russia is crucial and should be regarded as model studies providing the most representative insights into the structure, chorology, and dynamics of Arctic biota. This underscores the leading role of domestic scientists in assessing the biodiversity status of the Arctic while simultaneously placing a special responsibility on Russia in the practice of conserving and restoring the unique biocomponents of high-latitude ecosystems [83].

Against the backdrop of a relatively low species richness of Arctic biota as a whole, certain insect taxa (especially Diptera, Hymenoptera, Coleoptera) maintain relatively high diversity levels extending to the northern boundaries of the tundra. For instance, bumblebees serve as important ecological components of natural ecosystems as pollinators of entomophilous plants [84]. In the Arctic zone, this is a key group of pollinators, without which the biocenoses of these areas cannot be preserved in their natural state [84]. The impact of pesticides on such fragile ecosystems will result in a radical restructuring of biocenotic relationships in new agroecosystems, further exacerbating biodiversity impoverishment and leading to a corresponding decline in trophic chains, thus resulting in the loss of ecological self-regulation in these communities. Additionally, an increase in the frequency of forest fires in high-latitude forests is predicted and already observed [85,86], which will inevitably affect insect biomass.

Special concern among experts is currently caused by the rapid expansion of the ranges of several dominant arthropod species, which is attributed to climate change [e.g., [87,88,89,90]]. Climate factors can benefit certain insect species by increasing the available habitat area, while for others, they may have a negative impact. The ranges of serious pests of agricultural crops, such as *Leptinotarsa decemlineata*, *Helicoverpa armigera* (Hübner, 1805), *Phthorimaea operculella* (Zeller, 1873), *Eurygaster integriceps* Puton, 1881, *Frankliniella occidentalis* (Pergande, 1895), and others, are extended. These species with broad ecological plasticity are characterised by accelerated formation of a wide range of ecological adaptations to all types of abiotic and biotic environmental changes, including anthropogenic factors, and the ability to actively infiltrate plant communities. In the absence of competitors, outbreaks of these pests can lead to increased damage. The most reliable and powerful defenders of the forest are ants of the genus *Formica* s. str. During pest outbreaks, ants completely switch to them as the main prey and protect stands from significant damage and death [91]. However, in high latitudes, the species diversity and the number of ants is noticeably reduced. Only a few species are found in Russia beyond the Arctic Circle [92]. The northernmost species in Eurasia is *Leptothorax acervorum* (Fabricius, 1793), which in Russia reaches almost the shores of the Arctic Ocean in its distribution. However, this species has small colonies and does not play a significant role as an entomophagous. The main role is currently played by representatives of the genera *Formica* (*Serviformica*) and *Myrmica* in high latitudes. The role of species from these genera as biological agents is currently not so great in high latitudes. However, already now we are seeing a noticeable expansion of the ranges of some species and their numbers in the northern direction. Potentially, these species will play a significant role, on the one hand contributing to an increase in the harmfulness primarily of aphids (which have great biodiversity in the North [93] and potentially high harmfulness to cultivated plants) both greenhouse and open-field cultivation practices, reducing the effectiveness of specialized entomophagous. On the other hand, ants may be effective entomophagous in the future. In Siberian forests, currently Red forest ants (Formica s. str.) are dominant. They have no competitors among other invertebrates, effectively use feeding areas, easily switch from one prey to another (including both forest and agricultural pests). Therefore with a change in zoning with climate change, their role may be significant when using agricultural practices in the far north.

Some studies already suggest that the Russian Federation may benefit from climate change in the future (for example, longer growing seasons and warmer temperatures on the north, which could potentially lead to increased agricultural productivity; as the Arctic ice melts due to rising temperatures, new shipping trade routes and increased accessibility to natural resources, such as oil, gas, and minerals, become available), and this should be taken into account in strategic planning, but it should be done without disregarding scientific evidence [94]. The prospects for agriculture in northern regions are linked to the incredible rate of climate change that will affect regional land use [30,95,96]. Recent estimates suggest that extending the growing season will expand northern agriculture and introduce new crops that have historically been grown in warmer regions [97,98]. Engaging in agriculture in polar regions of Russia can be a challenging task due to harsh weather conditions and insufficient heat supplies. However, with the right approaches and technologies, agricultural crops can be successfully grown in these conditions. For instance, the use of greenhouses can protect crops from severe weather conditions and extend the growing season [77]. Furthermore, it is important to select crops wisely, choosing those that are well-suited for the local climate and growing conditions. Proper soil preparation can be crucial for cultivating agricultural crops in harsh regions. In polar areas, the amount of sunlight is limited during winter months, which can restrict the growth of agricultural crops. Artificial lighting systems can be utilised to provide plants with the necessary light for photosynthesis and growth.

Implementing sustainable agricultural practices, such as composting, crop rotation, and reducing pesticide use, to improve soil condition, conserve natural resources, and minimise the risk of pollution, can yield positive outcomes. These strategies can assist agriculture in harsh conditions, including polar regions of Russia, and contribute to the sustainable development of agriculture. Thus, moving the agricultural frontier northward will counterbalance the decline in food production in the temperate belt [30]. The northern regions of the Eurasian continent are already experiencing an increase in the sum of active temperatures, which creates more favorable conditions for growing crops; thus, the usual greenhouse agriculture of the Arctic regions will be gradually replaced by open-field cultivation practices [95,99]. The expansion and intensification of agriculture in the Arctic may cause serious problems related to biodiversity, ecosystem services, and biological invasions [100,101]. In the near future, it is necessary to develop a strategic plan for mitigating the consequences of climate change and adapting to them, based on the best available scientific data rather than reactionary actions.

However, it is important not to forget that the vulnerability of non-target insects to pesticides in the context of expanding agriculture in harsh conditions is a significant issue that requires attention. In these challenging conditions, insect pests can cause substantial damage to crops and reduce agricultural productivity. Insects in harsh environments may also be more susceptible to insecticides due to factors such as limited access to food and habitat, which can increase their susceptibility to chemical treatments [102]. At the same time, these conditions can also hinder the use of alternative pest control methods, such as biological control and integrated pest management, which rely on the presence of beneficial insects and other natural predators.

We consider that a good solution would be the introduction of “Integrated pest management”, where the emphasis is on pest control, not on their eradication. Agricultural practices in the north of Russia require sparing methods to maximise the conservation of the entomophage population, since many of them may suffer more from the introduction of insecticides, because pests can demonstrate resistance to insecticides and these are often species with a wide ecological valence [28]. There are theoretical developments for the use of Coccinellidae, Chrysopidae, and Syrphidae against sucking insects (Aphidoidea and Coccidae) in the greenhouse [103]. At the first stage of agricultural intensification, these practices can be successfully used. With climatic changes, the share of crops grown in the greenhouse will decrease, and theoretical developments of the use of specialized entomophagous in the open-field cultivation practices are already required. At this stage, the main limitations to the acclimatization of such entomophagous are the need to take into account photoperiodic reactions (due to the long light period during the growing season of crops) and the need to determine the cumulative temperature for the effective development of entomophagous insects in the far north [104]. With climate change, these restrictions will weaken over time. In the present period and in the near future, we consider that the role of agrotechnical practices in the open-field cultivation practices (as part of integrated pest management) will not only continue, but also increase.

Therefore, it is important to consider the vulnerability of insects to insecticides when expanding agriculture in harsh conditions and find ways to balance the need for effective pest control with the necessity of protecting human health and the environment. This may involve promoting the use of alternative pest control methods, expanding access to information on safe and effective pest control practices, and engaging the public in discussions on safe and responsible insecticide use. There is a need for scientific-based adaptive land development policies in the north in order to avoid a repeat of unsuccessful farming practices that have occurred in temperate and tropical regions [30,97,99,100,105]. By addressing these issues, insecticides can be used safely and effectively while protecting human health and the environment.

## 5. Successful Methods of Sustainable Agriculture

Human economic well-being, seemingly, will always take precedence over nature conservation until we reach a turning point where the destruction of the biosphere begins to threaten the existence of our species. The interrelation between human prosperity and the preservation of nature, especially insects, requires us, above all, to reach a societal consensus regarding the significance of insects. Only then will the development and implementation of new methods for sustainable agriculture become widely adopted worldwide. One of the key components of sustainable agriculture is the gradual reduction of pesticides. This principle is outlined in the EU’s “Farm to Fork Strategy”, a food program that aims to halve pesticide use on European agricultural lands by 2030 [106]. Positive shifts in this direction are being observed in Russia, as research is being conducted in favour of “ecologising” plant protection [e.g., [107,108,109,110]]. However, worldwide, especially in developing countries, such trends are not evident as there are limited technological capabilities to phase out the most hazardous methods of industrial agriculture. Moreover, the majority of biodiversity hotspots are located precisely in these countries.

The strategy of “win-win policy” [111] pertains to a strategy that would allow balancing the needs of agricultural production with the protection of ecosystems, particularly insects. Considering the potential impact of insecticides and pesticides on the environment, there is an opportunity to reduce the risk of environmental damage while ensuring continuous crop growth. Furthermore, by implementing sustainable agricultural practices and employing integrated pest management methods, it is possible to minimise the use of harmful chemical substances. By adopting such an approach, Russia can ensure the long-term viability of its agriculture while maintaining ecological sustainability. It can also lead to more sustainable utilisation of natural resources such as water and soil, and contribute to the creation of healthy and resilient ecosystems. For the agricultural sector, a “win-win policy” can result in increased productivity, reduced costs, and enhanced market competitiveness, while providing a more stable and predictable production environment.

However, implementing a “win-win policy” in agriculture also requires significant investments and changes in existing farming methods and systems. Governments, organisations, and the private sector need to work together to provide the necessary support and resources for implementing these changes. Additionally, it is crucial to ensure that farmers have the necessary knowledge, skills, and incentives to adopt sustainable agricultural practices. Therefore, the policy should be aimed at promoting sustainable agricultural practices. Examples include the use of environmentally friendly methods such as conservation tillage, integrated pest management, and cover crops to reduce the reliance on pesticides and fertilisers. Other strategies involve promoting agroforestry, which combines agriculture and forestry to produce food and other products while preserving natural ecosystems and habitats. At the initial stages, this may require significant efforts to attract investments in new technologies and practices and could be challenging to implement in traditional farming systems due to people’s unfamiliarity and lack of trust in new methods. The successful implementation largely depends on political and regulatory support, as the effectiveness of the strategy may be undermined by short-term economic interests and limited resources for research and development. This requires a long-term perspective and commitment to the adoption of new and environmentally friendly methods.

**Figure 3:**
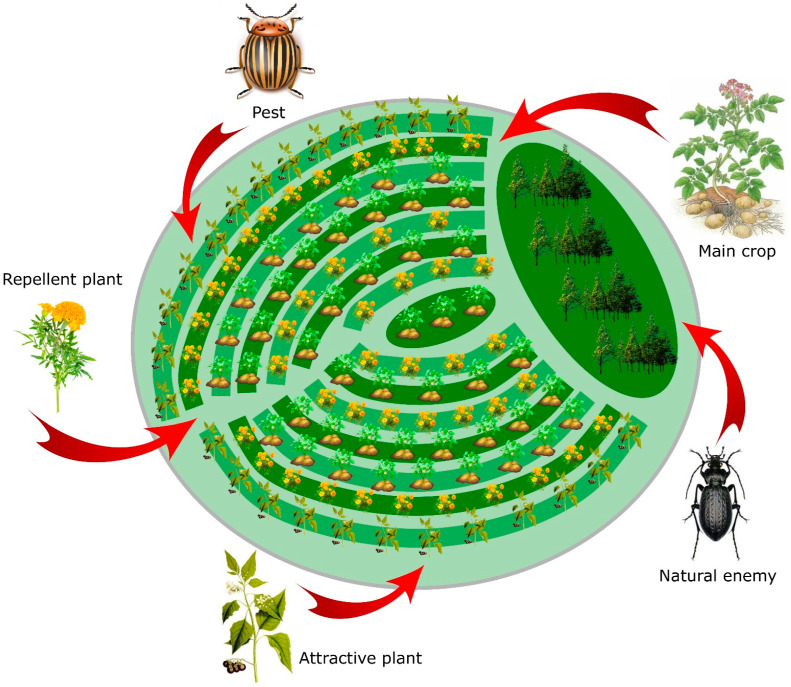
Schematic representation of “push-pull” technology in the conditions of northern Russia.

There are already successful examples of sustainable agriculture practices without the use of chemicals. One of the most successful applications, both economically and environmentally, is the “push-pull” technology (Figure 3) [112]. This method is based on the use of semi-chemicals released by the leaves and/or roots of specifically selected plants [113] to manipulate the behaviour of pests by attracting them through an attractant plant and repelling them with a repellent plant. This reduces the damage caused by pests and also enhances crop yield and soil quality [114,115]. The combination of cultivated plants in field conditions with non-cultivated companion plants, which attract natural enemies of pests, contributes to an increase in the population of these enemies [116,117]. Such non-cultivated companion plants can serve as an additional food source for natural enemies, providing them with pollen and/or nectar [118,119]. For example, planting native flowering plants near blueberry fields increased the abundance of entomophagous insects [120]. Similarly, floral strips planted around greenhouses increased the influx of entomophagous insects into the greenhouses and reduced the need for insecticides by enhancing biological control [121,122]. Hedges can also perform similar functions, albeit to a lesser extent [123]. Since agroecosystems lack sufficient resources to naturally control pests, they need to be artificially adapted to the needs of natural enemies in order to establish a relative balance and reduce pest populations [124,125]. Methods such as reduced tillage and conservation tillage can enhance pest control [126,127] and mitigate the negative consequences of landscape change [128]. Therefore, research should focus on understanding how soil management practices interact with landscape characteristics and influence plant protection. Landscape heterogeneity typically has a positive effect on the abundance and diversity of natural enemies [129,130,131]. Areas with natural vegetation can benefit natural enemies by providing refuge from insecticides, alternative food resources, and overwintering sites.

In practice, this strategy has already resulted in increased crop yields with a return on investment of over 2.2 times compared to 0.8 times for monoculture and just under 1.8 times for pesticide use [114,115]. The use of “pull and push” may be relevant to test the effectiveness in the conditions of the north of Russia with local plant species in the context of the fact that open-field cultivation practices will begin to displace the usual greenhouse agriculture and in the context of the expansion of insect pest areas to the north as the climate changes.

## 6. Legal Regulation of the Use of Pesticides

One of the challenges in achieving a balance between economic development and environmental conservation is the regulation of pesticide use. In developing countries, there is a lack of effective legal framework to regulate pesticide use or enforcement may be weak [132]. This can lead to excessive use of these chemical substances, which can have negative impacts on both the environment and human health. Additionally, the availability of alternative pest control methods, such as biological control, may be limited in these regions. This can further reinforce dependence on pesticides and contribute to their excessive use, as is the case in Russia [28].

Different countries have different rules and standards regarding insecticides, which can pose challenges for companies seeking to sell their products internationally. In the United States, the Environmental Protection Agency (EPA) is responsible for regulating and approving the use of insecticides under the Federal Insecticide, Fungicide, and Rodenticide Act (FIFRA) [133]. In the European Union, the European Food Safety Authority (EFSA) assesses the safety of insecticides, which are then approved for use by the European Commission [134]. China has adopted regulations on the handling of pesticides. According to the regulations, the Ministry of Agriculture is responsible for the management of pesticides throughout the chain, including production permits, risk assessment and registration, sales permits, monitoring and evaluation of registered products, and so on [135,136]. In Brazil, pesticides are regulated by the Brazilian Agency for Health Regulation (ANVISA), the Ministry of Agriculture, Livestock, and Supply (MAPA) and the Brazilian Institute of Environment and Renewable Natural Resources (IBAMA) [137]. In Russia, the use of pesticides is regulated by several federal laws, including the Federal Law “On the Safe Handling of Pesticides and Agrochemicals” and the Federal Law “On Plant Quarantine” [138]. These laws establish requirements for the registration, production, transportation, storage, use, and disposal of pesticides. The Federal Service for Supervision of Natural Resources (Rosprirodnadzor) is responsible for supervising compliance with these laws, while the Federal Service for Veterinary and Phytosanitary Surveillance (Rosselkhoznadzor) regulates the use of pesticides in agriculture. In addition, the Ministry of Agriculture and the Federal Service for Environmental, Technological, and Nuclear Supervision (Rostekhnadzor) also participate in pesticide regulation. The process of registering new insecticides can be slow and bureaucratic, delaying the introduction of new and potentially more effective products. The available information on the potential impact of insecticides on human health and the environment may be limited, making it difficult for regulatory bodies to make well-informed decisions regarding the safety of these products. Despite the existence of regulatory acts, a lack of enforcement can lead to the illegal use of insecticides and non-compliance with safety standards. Furthermore, in Russia, insufficient public participation in the regulation of insecticide use is often observed, which can result in a lack of understanding and support for regulatory measures.

To address these issues, it is important to promote international harmonisation of regulatory acts, streamline the registration process, enhance accessibility of information regarding the impact of insecticides, strengthen enforcement mechanisms, and engage the public in insecticide regulation. By tackling these challenges, insecticides can be used safely and effectively, while safeguarding human health and the environment. In Russia, for example, the Federal Law “On Organic Production” with amendments is in effect, which prohibits the use of agrochemicals, pesticides, antibiotics, growth stimulants, animal feed additives, and hormonal preparations, except those allowed by current national, interstate, and international organic production standards in the Russian Federation. It encompasses the use of biological control agents to combat pests and plant and animal diseases, as well as implementing measures to prevent losses caused by plant pests or plant products, based on protection against entomophagous (natural enemies of plant pests), selection of plant species and varieties, crop rotation, optimal plant cultivation methods, and thermal treatment methods of organic products [139]. Additionally, the Federal Law “On Technical Regulation”, establishes mandatory requirements for objects of technical regulation (products or goods) and related requirements for design processes (including research), production, construction, installation, commissioning, operation, storage, transportation, sales, and disposal. The requirements are applied for the purpose of: protecting the life or health of citizens, the property of individuals or legal entities, state or municipal property; protecting the environment, the life or health of animals and plants; preventing actions that mislead buyers, including consumers themselves; ensuring energy efficiency and resource conservation [140].

Legislative mechanisms and effective enforcement can play a crucial role in promoting environmentally responsible practices in sustainable agriculture by establishing standards for agricultural production and pesticide use, as well as holding companies accountable for their actions. Laws and regulatory acts can incentivise farmers to adopt sustainable methods and penalise those engaging in harmful activities, such as excessive pesticide use or soil degradation. The legal framework should also provide financial support for research and development of sustainable agricultural technologies. This could include tax incentives for farmers implementing environmentally friendly soil treatment methods or subsidies for agroforestry programs that promote the integration of trees into agricultural landscapes. Furthermore, the legal framework can facilitate measures to protect endangered species and their habitats, as well as prevent the spread of harmful invasive species. By working in collaboration with other stakeholders, the legal framework can help create a more sustainable and environmentally responsible agricultural sector that benefits both farmers and the environment [141,142,143].

**Figure 4:**
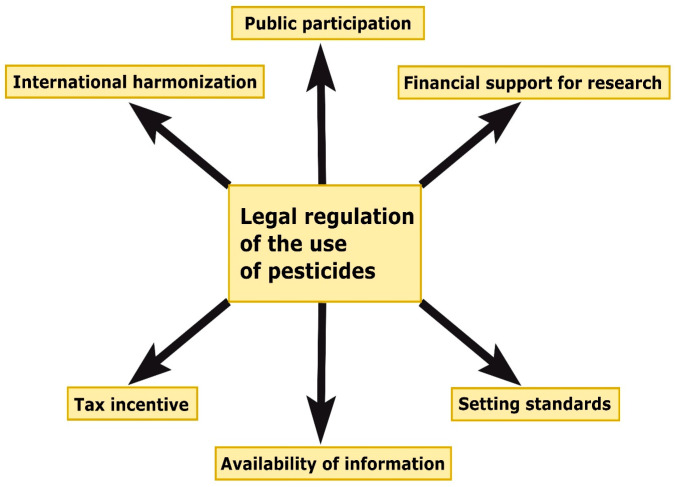
The main components for the successful legal regulation of the use of pesticides.

Ultimately, the preservation of insect populations in the context of agriculture requires a multifaceted approach that combines legal protection, economic incentives, as well as education and awareness (Figure 4). The legal system is called upon to play a crucial role in these efforts by providing the necessary foundation and support for the implementation of sustainable agricultural practices that promote the health of insect populations and ecosystems.

## 7. Conclusions

In conclusion, it should be noted that the expansion of agriculture in regions with harsh conditions is associated with a complex set of problems that require careful solutions. By balancing the need to protect agricultural crops with the necessity of safeguarding ecosystems and biodiversity, sustainable agriculture can be promoted in these regions. However, achieving this goal requires an interdisciplinary approach that takes into account the intricate interplay of ecological, economic, and social factors. Sustainable agriculture requires a long-term perspective and the development of scientifically sound methods for farming without the use of chemicals. It is essential to plan agricultural intensification in a way that preserves existing biodiversity, especially insects, as they play a crucial role in maintaining ecological balance in the biosphere. To conserve insects, for instance, we can support natural or restored areas within or near fields using methods like “push-pull”. On each specific site, we need to consider whether intensive farming would have a more detrimental impact on biodiversity and/or insect populations.

By replicating successful examples of sustainable and ecologically sound land development and implementing preventive measures, we can avoid negative consequences and environmental losses. Furthermore, considering the legal aspect of agroecological planning is crucial to ensure compliance with national and regional environmental policies. By adopting a responsible and scientific approach to land development, Russia can preserve the ecological health of its land for future generations.

## Data Availability

Not applicable.
